# Long-Lasting Sparks: Multi-Metastability and Release Competition in the Calcium Release Unit Network

**DOI:** 10.1371/journal.pcbi.1004671

**Published:** 2016-01-05

**Authors:** Zhen Song, Alain Karma, James N. Weiss, Zhilin Qu

**Affiliations:** 1 The UCLA Cardiovascular Research Laboratory, David Geffen School of Medicine, University of California, Los Angeles, Los Angeles, California, United States of America; 2 Department of Medicine (Cardiology), David Geffen School of Medicine, University of California, Los Angeles, Los Angeles, California, United States of America; 3 Department of Physics, Northeastern University, Boston, Massachusetts, United States of America; 4 Department of Physiology, David Geffen School of Medicine, University of California, Los Angeles, Los Angeles, California, United States of America; University of Virginia, UNITED STATES

## Abstract

Calcium (Ca) sparks are elementary events of biological Ca signaling. A normal Ca spark has a brief duration in the range of 10 to 100 ms, but long-lasting sparks with durations of several hundred milliseconds to seconds are also widely observed. Experiments have shown that the transition from normal to long-lasting sparks can occur when ryanodine receptor (RyR) open probability is either increased or decreased. Here, we demonstrate theoretically and computationally that long-lasting sparks emerge as a collective dynamical behavior of the network of diffusively coupled Ca release units (CRUs). We show that normal sparks occur when the CRU network is monostable and excitable, while long-lasting sparks occur when the network dynamics possesses multiple metastable attractors, each attractor corresponding to a different spatial firing pattern of sparks. We further highlight the mechanisms and conditions that produce long-lasting sparks, demonstrating the existence of an optimal range of RyR open probability favoring long-lasting sparks. We find that when CRU firings are sparse and sarcoplasmic reticulum (SR) Ca load is high, increasing RyR open probability promotes long-lasting sparks by potentiating Ca-induced Ca release (CICR). In contrast, when CICR is already strong enough to produce frequent firings, decreasing RyR open probability counter-intuitively promotes long-lasting sparks by decreasing spark frequency. The decrease in spark frequency promotes intra-SR Ca diffusion from neighboring non-firing CRUs to the firing CRUs, which helps to maintain the local SR Ca concentration of the firing CRUs above a critical level to sustain firing. In this setting, decreasing RyR open probability further suppresses long-lasting sparks by weakening CICR. Since a long-lasting spark terminates via the Kramers’ escape process over a potential barrier, its duration exhibits an exponential distribution determined by the barrier height and noise strength, which is modulated differently by different ways of altering the Ca release flux strength.

## Author Summary

Calcium (Ca) sparks, resulting from Ca-induced Ca release, are elementary events of biological Ca signaling. Sparks are normally brief, but long-lasting sparks have been widely observed experimentally under various conditions. The underlying mechanisms of spark duration or termination and the corresponding determinants remain a topic of debate. In this study, we demonstrate theoretically and computationally that normal brief sparks are excitable transients, while long-lasting sparks are multiple metastable states emerging in the diffusively coupled Ca release unit network, as a result of cooperativity and release competition among the Ca release units. Termination of a long-lasting spark is a Kramers’ escape process over a potential barrier, and the spark duration is the first-passage time, exhibiting an exponential distribution.

## Introduction

Calcium (Ca) is a ubiquitous signaling ion in biology, regulating both normal biological pathways as well as disease processes [1–3]. Besides biological signal transduction, Ca is required for muscle contraction and plays a key role in generating both normal and abnormal cardiac rhythms [4,5]. Intracellular Ca is stored in endoplasmic reticulum (ER) or sarcoplasmic reticulum (SR) membrane networks exhibiting complex structures within the cell. Ca is released from these internal stores via Ca release channels, called IP_3_ receptors (IP_3_Rs) or ryanodine receptors (RyRs). IP_3_Rs or RyRs are clustered on the membrane of the ER/SR, forming discrete Ca release units (CRUs). Opening of IP_3_Rs or RyRs is sensitized by cytoplasmic Ca, forming a positive feedback loop called Ca-induced Ca release (CICR). CICR causes the IP_3_Rs or RyRs to open and close collectively in a cluster, resulting in random and discrete Ca release events, called Ca sparks [6]. A spark can be elicited by Ca entry from sarcolemmal Ca channels or occur spontaneously via CICR. Ca sparks have been considered as the dynamical elements which interact to generate sub-cellular and cellular dynamics for Ca signaling and muscle contraction, such as Ca waves and oscillations [7–12] and more complex nonlinear dynamics in the heart [5,13–18].

**Fig 1:**
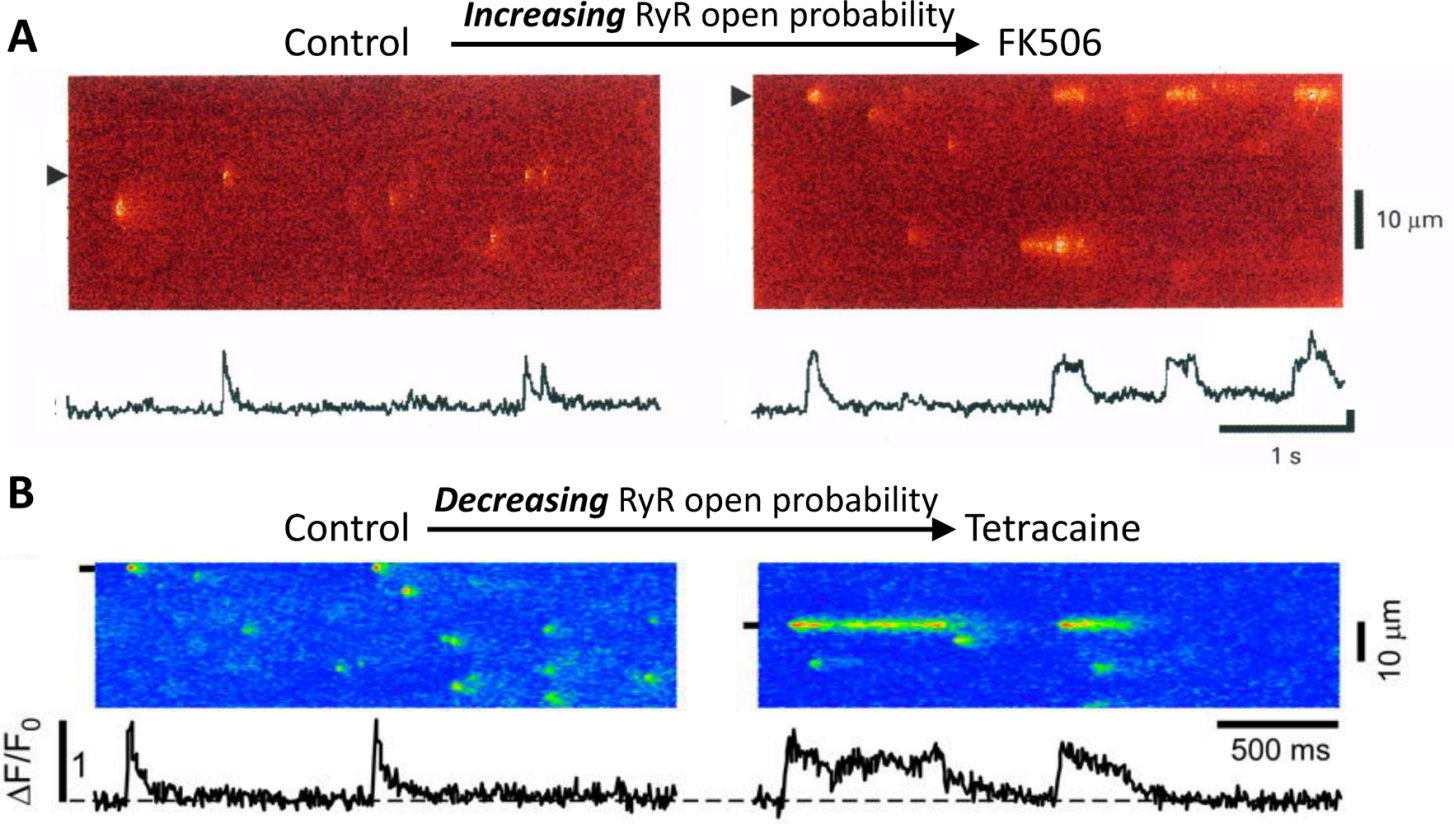
Examples of transition from normal short sparks to long-lasting sparks observed in experiments. A. Increasing RyR open probability by FK506 promoted long-lasting sparks. Modified from Xiao et al [20]. B. Decreasing RyR open probability by tetracaine promoted long-lasing sparks. Modified from Zima et al [27].

A Ca spark normally lasts for 10 to 100 ms, but long-lasting sparks with duration of several hundred milliseconds to seconds have been widely observed in different types of cells under various conditions [19–28]. Paradoxically, the transition from normal sparks to long-lasting sparks can be induced both by agents that increase RyR open probability [19–24] (e.g., Fig 1A) and agents that decrease RyR open probability [25–27] (e.g., Fig 1B). Long-lasting sparks have also been observed near the nucleus in ventricular myocytes without altered RyR open probability [28]. Xiao et al [20] proposed a single channel mechanism for long-lasting sparks induced by FK506 or rapamycin by showing that FK506 resulted in prolonged subconductance open states of RyRs. However, it has also been shown that FK506 or rapamycin disrupt the coupled gating of RyRs, substantially shortening their open time [29], contrary to prolongation of spark duration by FK506. Moreover, reducing RyR open probability by tetracaine or Mg^2+^ does not increase the open time, but still can result in long-lasting sparks [25,27]. In a simulation study using a single CRU model with network SR (NSR) Ca concentration held constant, Sobie et al [30] showed that decreasing the cooperative effect of coupled gating (equivalently increasing RyR open probability) promoted long-lasting sparks, which was further verified theoretically by Hinch [31]. Hinch showed that the transition from normal sparks to long-lasting sparks occurs when the deterministic dynamical system governing CICR at the single CRU level changed from monostable to bistable. Using a similar single CRU model, Stern et al [32] showed that long-lasting sparks could be induced by increasing the intra-SR Ca diffusion rate or increasing RyR open probability. However, none of the single CRU studies have explained the experimental observations that reducing RyR open probability can also promote long-lasting sparks.

Although a spark is a collective behavior of the IP_3_Rs/RyRs clustered in a CRU, the CRUs in a cell are not isolated from each other, but are coupled via intra-SR and cytosolic Ca diffusion. Therefore, the spark behavior of a CRU also depends on the behaviors of the neighboring CRUs, which can only be understood in the context of the CRU network of the cell, rather than a single CRU. In this study, we propose a theory for the transition from normal brief sparks to long-lasting sparks based on a coupled CRU network, which unifies the seemingly contradictory experimental observations described above. We show theoretically and in computer simulations that when the Ca release flux of a CRU is low, CICR cannot be maintained at the single CRU scale, resulting in normal short sparks. When the Ca release flux is high, CRUs fire frequently, and the firing competition between neighboring CRUs prevents the CRUs from sustaining the CICR state, also resulting in normal short sparks. However, when the Ca release flux is in an intermediate range, Ca release is strong enough to maintain CICR but low enough to have a low spark frequency. Consequently, the quiescent neighboring CRUs provide the additional source of Ca to prevent SR depletion to a level causing CICR termination, thereby resulting in long-lasting sparks. From a fundamental nonlinear dynamics perspective, our findings reveal that different sparse patterns of long-lasting sparks correspond to different dynamical attractors of the CRU network. Those attractors are stable in the deterministic limit, where the spark duration is infinite, and metastable in the presence of RyR channel stochasticity, where long-lasting sparks terminate via Kramers-like escape across the barrier between the metastable firing state and the non-firing state. As a result of this escape process, the spark duration exhibits an exponential distribution determined by the height of the barrier and the noise strength.

## Results

### Ca spark dynamics in a ventricular myocyte model

**Fig 2:**
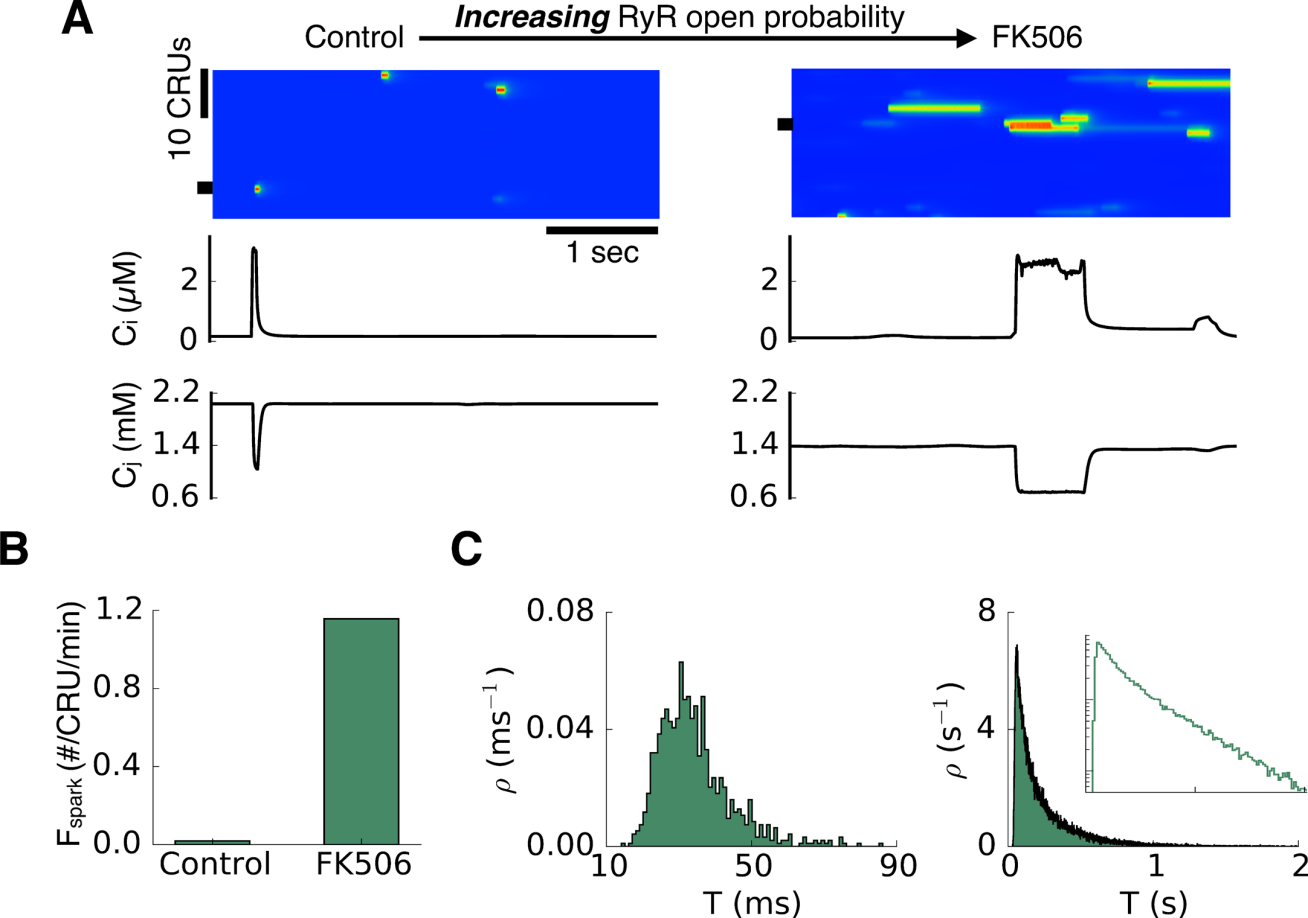
Long-lasting sparks promoted by increasing RyR open probability in a ventricular myocyte model. A. Line scans, cytosolic Ca (c_i_), and SR Ca (c_j_) from a CRU (marked by the black bar) for control and FK506. B. Spark frequency (F_spark_) under two conditions. C. Spark duration distributions for the two conditions. Inset in the second panel is a semi-log plot of the same data. The control parameters are: α = 4 and β = 20. Change from control to FK506 by decreasing β to 6.67.

We first carried out simulations using a ventricular myocyte model developed by Restrepo et al [14] with modifications (see Methods) to recapitulate the experimental observations of long-lasting sparks induced by ryanodine [21] or FK506 shown in Fig 1 [20]. Fig 2 shows a transition from short sparks to long-lasting sparks in the model when the RyR open probability was increased by prolonging the open time of the RyRs to simulate FK506 in Fig 1A. The SR Ca load (c_j_ in Fig 2A) under control conditions was ~2.0 mM and depleted to ~1.0 mM during a spark, while the SR load after simulated FK506 was reduced to ~1.4 mM and depleted to ~0.7 mM during a spark. The spark frequency (i.e., spark probability) became higher after RyR open probability was increased (Fig 2B), agreeing with the experimental observations [21]. The left panel and right panel in Fig 2C show the spark duration distributions before and after FK506, respectively. Under the control condition, the spark duration exhibited a bell-shaped (close to Gaussian) distribution. After FK506, the spark duration exhibited an exponential distribution. These properties also agree well with the experimental observations [20,21].

**Fig 3:**
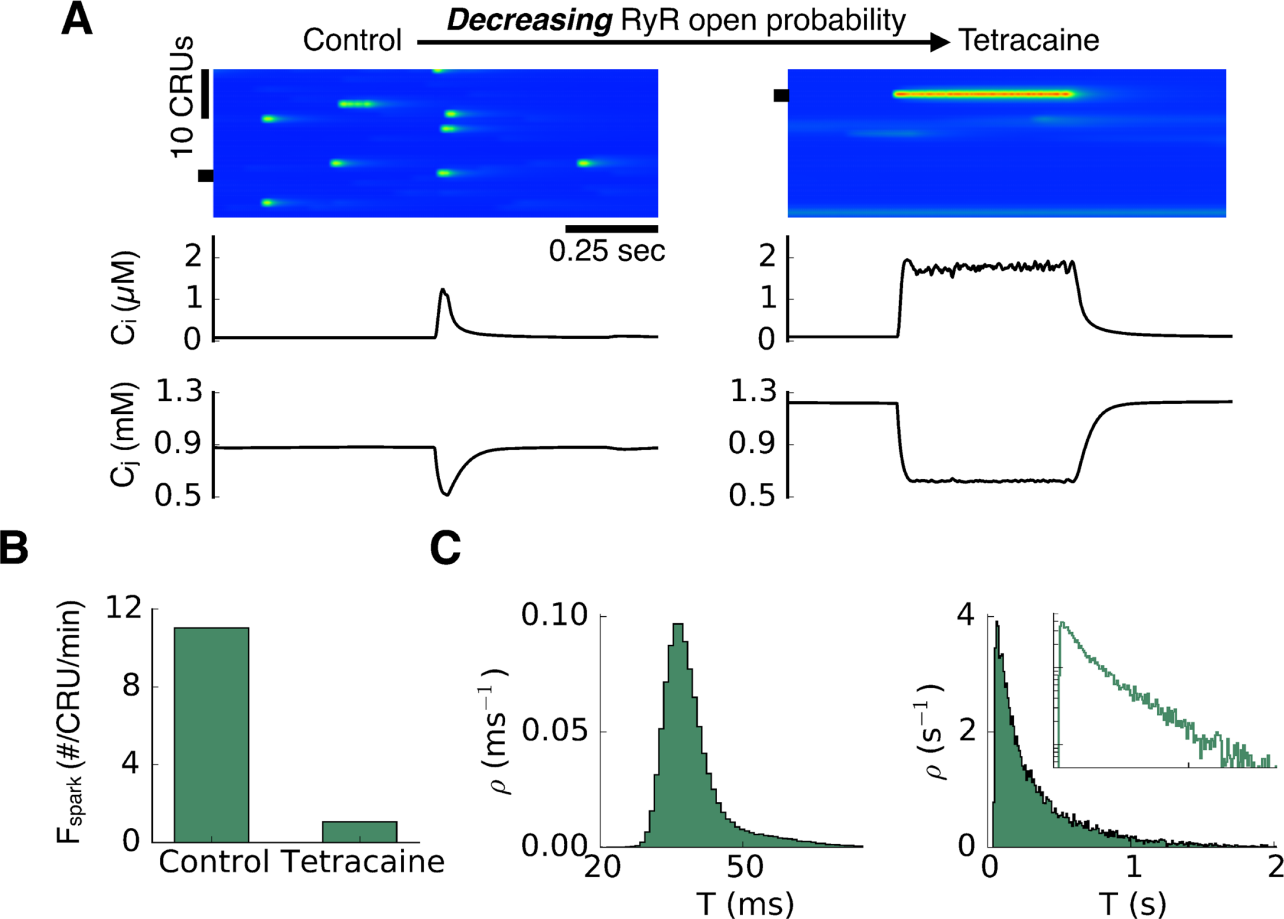
Long-lasting sparks promoted by decreasing RyR open probability in a ventricular myocyte model. A. Line scans, cytosolic Ca (c_i_), and SR Ca (c_j_) from a CRU (marked by the black bar) for control and tetracaine. B. Spark frequency under two conditions. C. Spark duration distributions for the two conditions. Inset in the second panel is a semi-log plot of the same data. The control parameters are: α = 4 and β = 1. Change from control to tetracaine by decreasing α from 4 to 1.

Fig 3 shows a transition from short sparks to long-lasting sparks when the RyR open probability was decreased by increasing the closed time of the RyRs to simulate the effects of tetracaine in Fig 1B [27]. In this case, the SR Ca load (c_j_ in Fig 3A) under control conditions was ~0.9 mM and depleted to ~0.5 mM during a spark, while the SR load after simulated tetracaine was increased to ~1.2 mM and depleted to ~0.6 mM during a spark. The spark frequency became lower after the RyR open probability was reduced (Fig 3B), also agreeing with experimental observations [33,34]. The spark duration distribution exhibited a bell-shaped distribution under control and became exponential after tetracaine (Fig 3C), which also agrees with the experimental observations [27].

**Fig 4:**
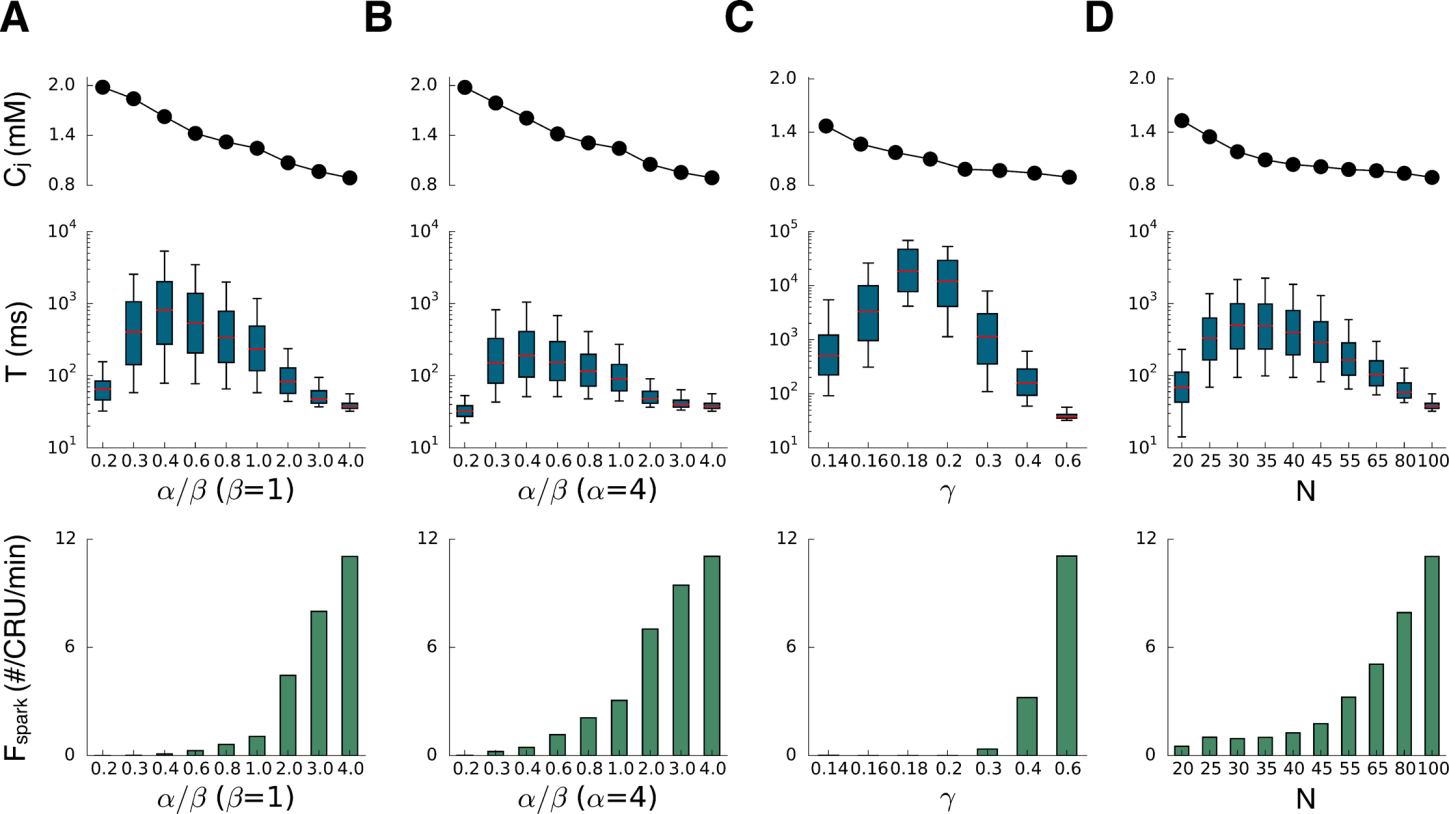
Effects of altering Ca release flux on spark dynamics. Top panels plot the SR Ca load measured by c_j_ which was the maximum value among all CRUs detected in the whole cell during simulation period after reaching the steady state. Middle panels plot the spark duration which are presented using box plots. The red line in each box is the median of spark duration. The bottom and top of the box are the first and third quartiles. We used 5^th^ percentile and 95^th^ percentile for the whiskers in each case, respectively. Bottom panels plot the spark frequency. **A**. Altering α to alter the RyR close time. **B**. Altering β to alter the RyR open time. **C**. Changing the single channel conductance of RyRs. **D**. Changing the number of RyRs in a CRU.

We then systematically investigated the effects of altering the Ca release strength of the CRUs on spark dynamics (Fig 4). We used four ways to alter the Ca release strength: the RyR closed-to-open rate constant (scaled by α, Fig 4A), the RyR open-to-closed rate constant (scaled by β, Fig 4B), the single channel conductance of RyR (scaled by γ, Fig 4C), and the number of RyRs in a CRU (RyR cluster size N, Fig 4D). We plotted the SR Ca load and spark duration using box plots for different release strength (Note: the x-axis is not a linear scale). Decreasing α, which decreases the RyR open probability by increasing the closed time of the RyRs (simulating tetracaine [27,33]), first increased and then decreased the spark duration. SR Ca load increased monotonically as α decreased (Fig 4A). Note that the spark duration was normal (short) at both low and high RyR open probability, while long-lasting sparks occurred at the intermediate range. Increasing β, which decreases the RyR open probability by shortening the open time of RyRs (simulating flecainide [33]), had the same effect as decreasing α, but the spark duration was shorter for the same RyR open probability (note: the steady-state RyR open probability is the same for the same α/β ratio, see Methods). We observed the same non-monotonic behavior of spark duration for altering the single channel conductance of RyR (Fig 4C) and the RyR cluster size (Fig 4D). In these two cases, despite the large change in spark duration, the SR Ca load only changed slightly. In all four cases, the spark frequency decreased as the Ca release flux strength was reduced.

The results in Fig 4 show that for some conditions, long-lasting sparks occur with an increase in RyR open probability, whereas for other conditions, they occur with a decrease. Indeed, the conditions before the RyR open probability was altered were different in Figs 2 and 3 where long-lasting sparks were induced by increasing and decreasing RyR open probability, respectively. Although the conditions were different as indicated by the baseline spark frequencies (Fig 2B and Fig 3B), the spark duration distributions under two conditions were similar (Figs 2C and 3C). When long-lasting sparks were induced by increasing the RyR open probability, the spark frequency increased (Fig 2B), while when long-lasting sparks were induced by decreasing RyR open probability, the spark frequency decreased (Fig 3B). These behaviors agree well with experimental results [21,26,27], supporting the modeling prediction that long-lasting sparks can be induced by different alterations of RyR properties starting from different initial states.

### A theory of long-lasting sparks

To elucidate the mechanism of long-lasting sparks, we developed a theory using coupled CRUs and carried out simulations to verify the theory.

Theory—According to previous studies [5,31,32], normal brief sparks occur when the corresponding deterministic limit of the CRU model is monostable, and long-lasting sparks occur when it is bistable. Here we use deterministic models of uncoupled single and coupled CRU systems to perform theoretical analyses by investigating their steady-state solutions.

**Fig 5:**
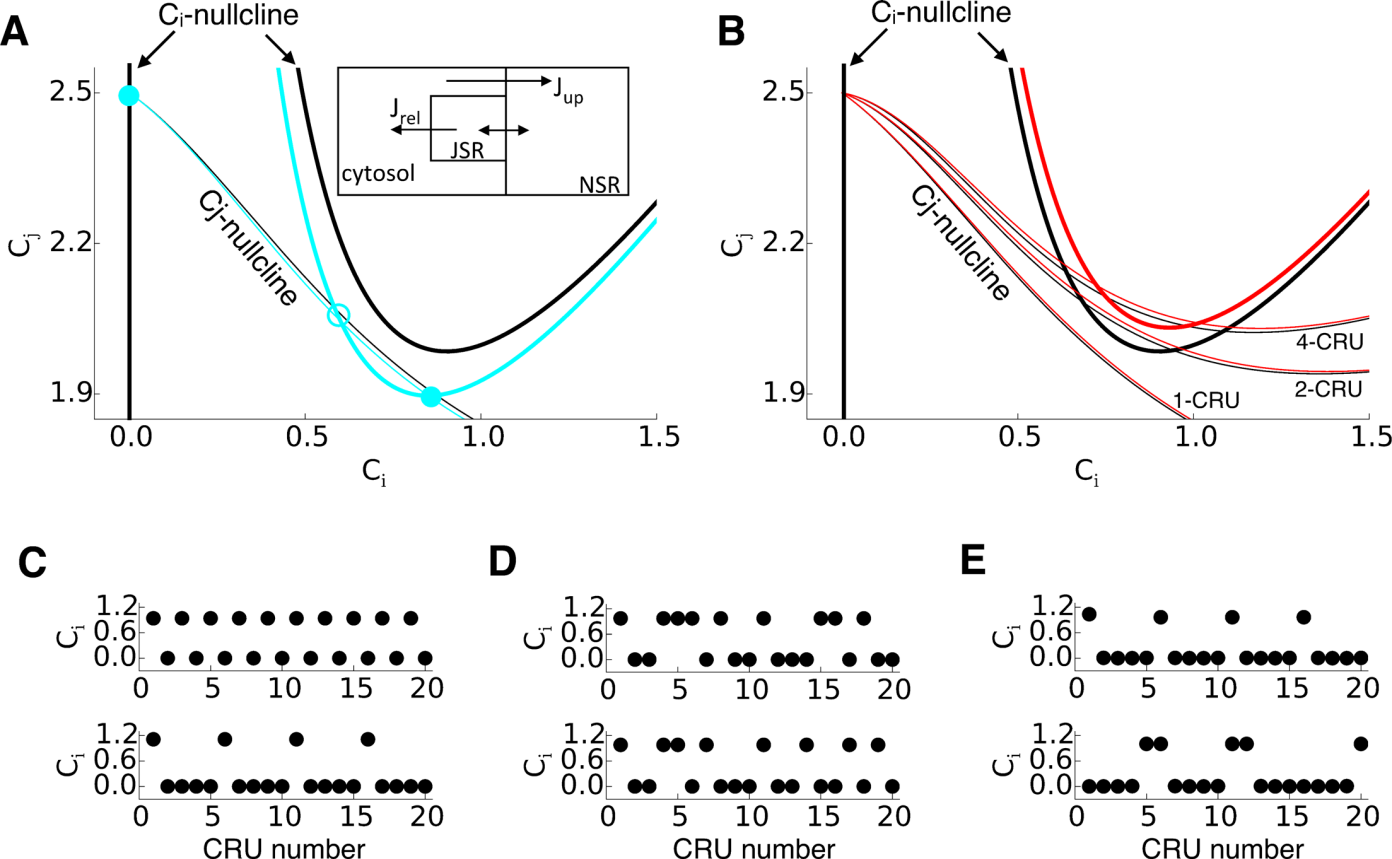
Nullcline structure of deterministic limit of spark dynamics. **A**. Nullclines and steady-state solutions of a single CRU. The thick lines represent the c_i_-nullcline (Eqs 4 and 5) and the thin lines represent the c_j_-nullcline (Eq 6). Black: α = 1. Cyan: α = 1.15. All the other parameters were kept the same: β = 1.2, c_0_ = 5, γN = 1.25, μ = 0.85, δ = 0.5, and D_NJ_ = 1.5. **B**. Nullclines and steady-state solutions of coupled CRUs. The parameters are the same as in A with D_NN_ = 0.5. Black: α = 1. Red: α = 0.93. The c_j_-nullclines for two (Eq 9) and more CRUs are labeled accordingly. **C**. Spatially periodic solutions in a 1D cable. **D**. Random spatial patterns in the same cable as in C. **E**. Spatially periodic and random patterns after cytosolic Ca diffusion was added with a diffusion constant 0.012.

Consider a single CRU model with three compartments indicated in Fig 5A. The differential equations describing the Ca dynamics are:

$$\frac{dc_{i}}{dt}=J_{rel}-J_{up}=\gamma Np(c_{j}-c_{i})-\frac{\mu c_{i}}{\delta +c_{i}}$$ (1)

$$\frac{dc_{j}}{dt}=-J_{rel}+J_{Nj}=-\gamma Np(c_{j}-c_{i})+D_{Nj}(c_{N}-c_{j})$$ (2)

$$\frac{dp}{dt}=\alpha c_{i}^{2}(1-p)-\beta p$$ (3)

where c_i_, c_j_ and c_N_ are Ca concentrations in cytosolic, junctional SR (JSR) and network SR (NSR), and p is the open probability of a single RyR. N is the number of RyRs in a CRU, γ is the single channel conductance of the RyRs, μ is the SERCA pump strength, δ is its half-saturation value, and D_Nj_ is the Ca diffusion constant from NSR to JSR. The RyR model (Eq 3) is described by a 2-state model with an open state and a closed state. The rate constant for closed-to-open is $\alpha c_{i}^{2}$ and the rate constant for open-to-closed is β. Instead of fixing c_N_ to a constant as in previous models [30–32], we assume that the total Ca of the CRU is constant, i.e., *c*_0_ = *c*_*N*_ + *c*_*i*_ + *c*_*j*_. We further assume that RyRs reach their steady-state rapidly, which implies that the c_i_-nullcline is the solution of Eq 1 for d*c*_*i*_/dt = 0 with $p_{s}=\frac{\alpha c_{i}^{2}}{\alpha c_{i}^{2}+\beta }$ (the thick black lines in Fig 5A), which yields:

$$c_{i}=0$$ (4)

and

$$c_{j}=c_{i}+\frac{\mu c_{i}}{(\delta +c_{i})\gamma Np_{s}}\;.$$ (5)

The c_j_-nullcline in turn is the solution of Eq 2 when d*c*_*j*_/dt = 0 (thin black line in Fig 5A), yielding:

$$c_{j}=\frac{\gamma Np_{s}c_{i}+D_{Nj}(c_{0}-c_{i})}{2D_{Nj}+\gamma Np_{s}}$$ (6)

The steady-state solutions (fixed points of the system) correspond to the intersections of those two nullclines. The system always has a steady-state solution according to Eqs 4 and 6: c_i_ = 0, p_s_ = 0, and c_j_ = c_0_/2. Additional steady-state solutions can be obtained using Eqs 5 and 6. Subtracting Eq 6 from Eq 5, one has

$$\Delta c_{j}=\frac{\mu c_{i}}{(\delta +c_{i})\gamma Np_{s}}-\frac{D_{Nj}(c_{0}-3c_{i})}{2D_{Nj}+\gamma Np_{s}}\;.$$ (7)

According to Eq 7, when Δ*c*_*j*_ > 0 holds for all c_i_>0, no additional intersections of the two nullclines and thus no additional steady states exist, so that the system is monostable. When this condition fails, new steady-states occur, leading to bistability in which CICR is maintained in the deterministic limit. It can be easily shown from Eq 7 that the condition Δ*c*_*j*_ > 0 for all c_i_>0 tends to fail by increasing c_0_, D_Nj_, or the Ca release flux strength (i.e., γNp_s_). As shown in Fig 5A, increasing RyR open probability moves the c_i_-nullcline downward but has a small effect on c_j_-nullcline (from black to cyan in Fig 5A), promoting bistability. Thus increasing RyR open probability or Ca diffusion from NSR to JSR tends to result in bistability for long-lasting sparks, agreeing with the previous studies [30–32]. However, if the CRU under control conditions is monostable, i.e., Δ*c*_*j*_ > 0 holds for all c_i_>0 then this will always hold for reduced γNp_s_. Therefore, reducing RyR open probability suppresses bistability and thus long-lasting sparks, which cannot explain the experimental observations and simulation results that reducing RyR open probability also promotes long-lasting sparks.

Next, we consider networks of coupled CRUs. First consider two coupled CRUs (Fig 5B). To facilitate analytical treatment, we assume no cytosolic Ca diffusion between the two CRUs, which are then only coupled via Ca diffusion in the NSR. The equations describing the variables of the second CRU are the same as Eqs 1–3 and an additional equation is needed for the coupled system to describe the Ca concentration in the NSR, i.e.,

$$\frac{dC_{N}}{dt}=D_{NN}(c_{N}-C_{N})+D_{Nj}(C_{j}-C_{N})+\frac{\mu C_{i}}{\delta +C_{i}}$$ (8)

where we denote the variables of the second CRU with capital letters and D_NN_ is the diffusion constant of Ca diffusion between the NSR of the two CRUs. The total Ca of the two coupled CRUs remains constant, satisfying: 2*c*_0_ = *c*_*N*_ + *c*_*i*_ + *c*_*j*_ + *C*_*N*_ + *C*_*i*_ + *C*_*j*_. Since the two CRUs are identical, the coupled system can always have a uniform steady state solution which is the same steady-state solution as the one of an isolated single CRU. However, it is possible that spatially non-uniform solutions exist. Assuming that the second CRU does not fire, staying at its steady state C_i_ = 0 (C_j_ = C_N_), then the c_i_-nullcline of the first CRU is unchanged, but the c_j_-nullcline of the first CRU becomes

$$c_{j}=\frac{\gamma Np_{s}c_{i}+D_{Nj}(2c_{0}-c_{i})/3}{4D_{Nj}/3+\gamma Np_{s}}$$ (9)

As shown in Fig 5B, the c_j_-nullcline (Eq 9) is elevated from that of a single CRU (Eq 6). This implies that when two monostable CRUs are coupled together, and only one of them fires, the system can become bistable, i.e., the unfired CRU provides an additional source of Ca via SR diffusion so that the SR of the firing CRU is not depleted to the level that terminates CICR. This bistability can be enhanced when the number of diffusively-coupled CRUs in the network is further increased (Fig 5B). Note that the number of CRUs needed for bistability still relies on RyR open probability or the Ca release flux strength as in the single CRU. For example, reducing RyR open probability elevates the c_i_-nullcline but has little effect on the c_j_-nullclines (red curves in Fig 5B).

**Fig 6:**
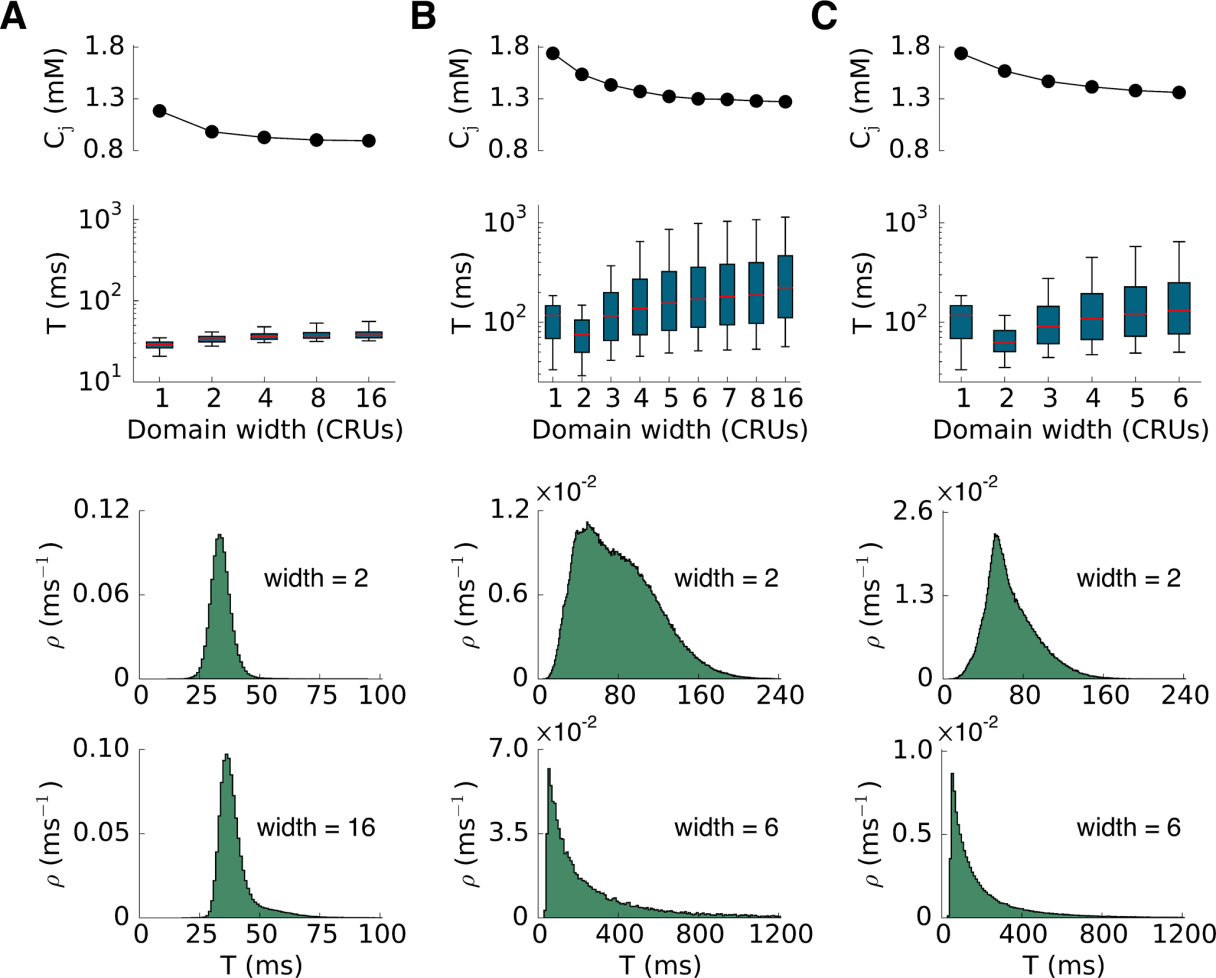
CRU intra-SR diffusive coupling promotes long-lasting sparks and variability of duration. Plotted in each case are SR Ca load, spark duration in box plots, a histogram of spark duration for 2x2x2, 6x6x6, and 16x16x16 domains as labeled. A. Parameters are the same as the control condition in Fig 3. B. Same as A but α was reduced from 4 to 1 (the tetracaine condition in Fig 3). C. Same as B but Ca diffusion in NSR was reduced by 2 fold, i.e., D_NN_→D_NN_ /2. The distribution of spark duration evolves from a narrow distribution for normal sparks to a broader exponential distribution reflecting a Kramers’ escape process for long-lasting sparks.

One can infer from the preceding theoretical analysis that in a network of coupled CRUs, different non-uniform steady-state solutions corresponding to different spatial firing patterns of sparks can exist in addition to the homogeneous monostable steady-state solutions, so that the network dynamics exhibits multi-stability. The specific pattern that the system selects depends on initial conditions. To test this prediction, we performed simulations in a one-dimensional cable of coupled CRUs with the single CRU described by Eqs 1–3. The parameters were kept the same as for the control (black curves) in Fig 5B. Fig 5C shows a spatially period-2 and period-5 solutions, while Fig 5D shows two different random spatial patterns in the cable. For the sake of theoretical treatment, we did not allow Ca diffusion in the cytosolic space in the theoretical analysis. If Ca diffusion is allowed in the cytosolic space, it becomes more difficult for the non-uniform steady-state solutions to form. This is because cytosolic Ca diffusion tends to synchronize spark firing, thereby favoring the spatially uniform state. For example, after we added Ca diffusion in the cytosolic space with a small diffusion constant, the spatially period-2 solution was no longer observed, but higher periods as well as random patterns with longer spatial scales could still exist (Fig 5E). Importantly, contrary to cytosolic Ca diffusion, enhancing intra-SR Ca diffusion promotes multi-stability (see results in Fig 6 below).

Based on the analysis above, we can make the following theoretical predictions for Ca sparks in coupled CRU networks. Differing from its deterministic limit, in which the dynamically selected spark patterns is dictated by the initial conditions, the CRU network system (in a real or model cell) exhibits spontaneous sparks which occur randomly in space and time. Therefore, the selection of a spark pattern depends on the spark firing rate. When the spark probability is high, many CRUs fire, and there are not enough unfired CRUs to allow Ca to diffuse via the NSR from the unfired to the fired CRUs to maintain CICR (corresponding to the black nullclines in Fig 5B). Thus the chance of forming a long-lasing spark is very low if not zero. As RyR open probability is reduced, the c_i_-nullcline moves upwards. However, since the spark probability also decreases, the c_j_-nullcline also moves upwards (this is different from a single CRU in which the c_j_-nullcline hardly moves) since the unfired CRUs provide additional Ca. As long as the c_j_-nullcline moves faster than the c_i_-nullcline does, the two can intersect to result in long-lasting sparks. Once a CRU fires as a long-lasting spark, the SR Ca in its neighboring CRUs is decreased. This suppresses the probability that neighboring CRUs will fire since the spark probability increases exponentially with SR Ca [30], thereby further stabilizing the long-lasting sparks. Stabilization is even more pronounced for faster SR Ca diffusion. As the RyR open probability decreases further, CRUs fire even less frequently. However, as a response to this decrease, the c_j_-nullcline will no longer move upwards as much as the c_i_-nullcline since adding more distal unfired CRUs does not help to elevate the c_j_-nullcline. Eventually, the two nullclines lose their intersections required for long-lasting sparks, and only the brief sparks occur. This theoretical framework allows us to interpret the computer simulation results shown in Fig 4. A reduction of the Ca release flux strength first promoted the transition from normal short sparks to long-lasting sparks but then further reduction of release flux caused a transition back to short sparks. Therefore, long-lasting sparks result from the balance between RyR open probability, which controls the spark frequency and hence the average ratio of firing and non-firing CRUs in the network, and intra-SR Ca diffusion that promotes long-lasting sparks in regions of the cell where this ratio is sufficiently low for a firing CRU to be surrounded by several non-firing CRUs. The multiplicity of firing patterns reflects different network configurations of firing and non-firing CRUs.

Computer simulations—To further confirm the theoretical predictions that CRU coupling and spark rate are two of the key factors in promoting long-lasting sparks, we carried out additional simulations using the same detailed stochastic CRU model as in Figs 2–4. First, we gradually coupled more and more CRUs together in a 3D geometry to demonstrate the coupling effect (Fig 6). When the parameters were set using the control conditions in Fig 3, adding more CRUs only slightly increased spark duration (Fig 6A), which all followed bell-shaped distributions. When the parameters were set using the conditions simulating tetracaine, spark duration was brief for small CRU networks, but increased dramatically with network size (Fig 6B). The spark duration distributions changed from bell-shaped distributions to exponential distributions. When the intra-SR Ca diffusion constant was reduced, the spark duration became shorter (Fig 6C). In other words, enhancing intra-SR Ca diffusion promotes long-lasting sparks.

**Fig 7:**
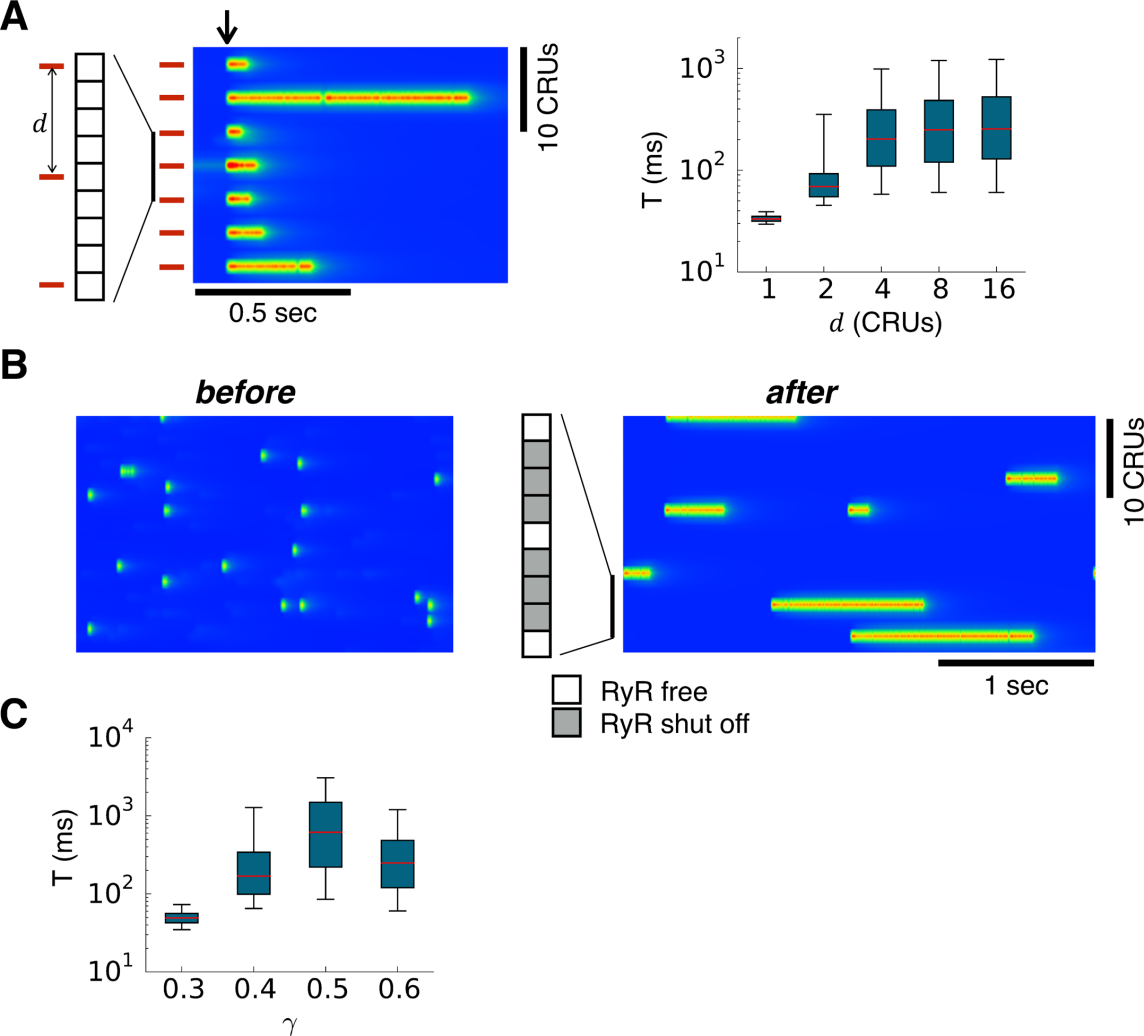
Effects of spark probability. **A**. Left panel is an example line scan showing stimulated sparks. Bars indicate the CRUs that were forced to fire and the arrow indicates the time when the stimulation was given. Stimulation of the CRUs was done by initially setting the RyRs in the CRU to the open state at the time indicated by the arrow. The right panel shows spark durations versus the spacing (denoted as d) between the stimulated CRUs. Simulations were carried out in the whole cell and the parameters were the same as in the tetracaine condition in Fig 3 in which the spontaneous spark rate is low. **B**. Line scans before (left) and after (right) a portion of the CRUs were turned off. 3 of every 4 CRUs were shut off as indicated in the inset. Simulations were carried out in the whole cell and the parameters were the same as in the control condition in Fig 3. **C**. Spark distribution versus single channel conductance of RyR for a fixed stimulation spacing. The conditions and simulations were the same as in A and the spacing between two stimulation sites was d = 8 CRUs.

We then carried out simulations to demonstrate the effect of spark rate on Ca spark dynamics. In the case of tetracaine, the RyR open probability was reduced, and the spark probability was low. To increase the spark rate, we applied stimuli periodically in space (bars in Fig 7A) to fire sparks at a certain time point after the system reached equilibrium (arrow in Fig 7A). When the stimuli were applied to all CRUs, the spark durations were brief as under normal control conditions. As the spatial distance between the stimulated sites increased, the averaged spark duration prolonged and saturated as the distance increased further. In another type of simulation, we used the control condition as in Fig 3 in which the spark rate was high and the spark duration was brief. To reduce the spark rate of the CRU network, we shut off the RyRs in a portion of the CRUs in the network, which caused long-lasting sparks to occur (Fig 7B).

Finally, we demonstrated the effect of changing the nullclines by reducing the RyR conductance. The simulations were the same as in Fig 7A, but we kept the distance between the stimulated sites at 8 CRUs. As shown theoretically, reducing γ makes the two nullclines move further apart and eventually lose their intersection required for bistability. As shown in Fig 7C, the spark duration first increased and then decreased, becoming brief when γ became small enough.

### Determinants of spark duration

**Fig 8:**
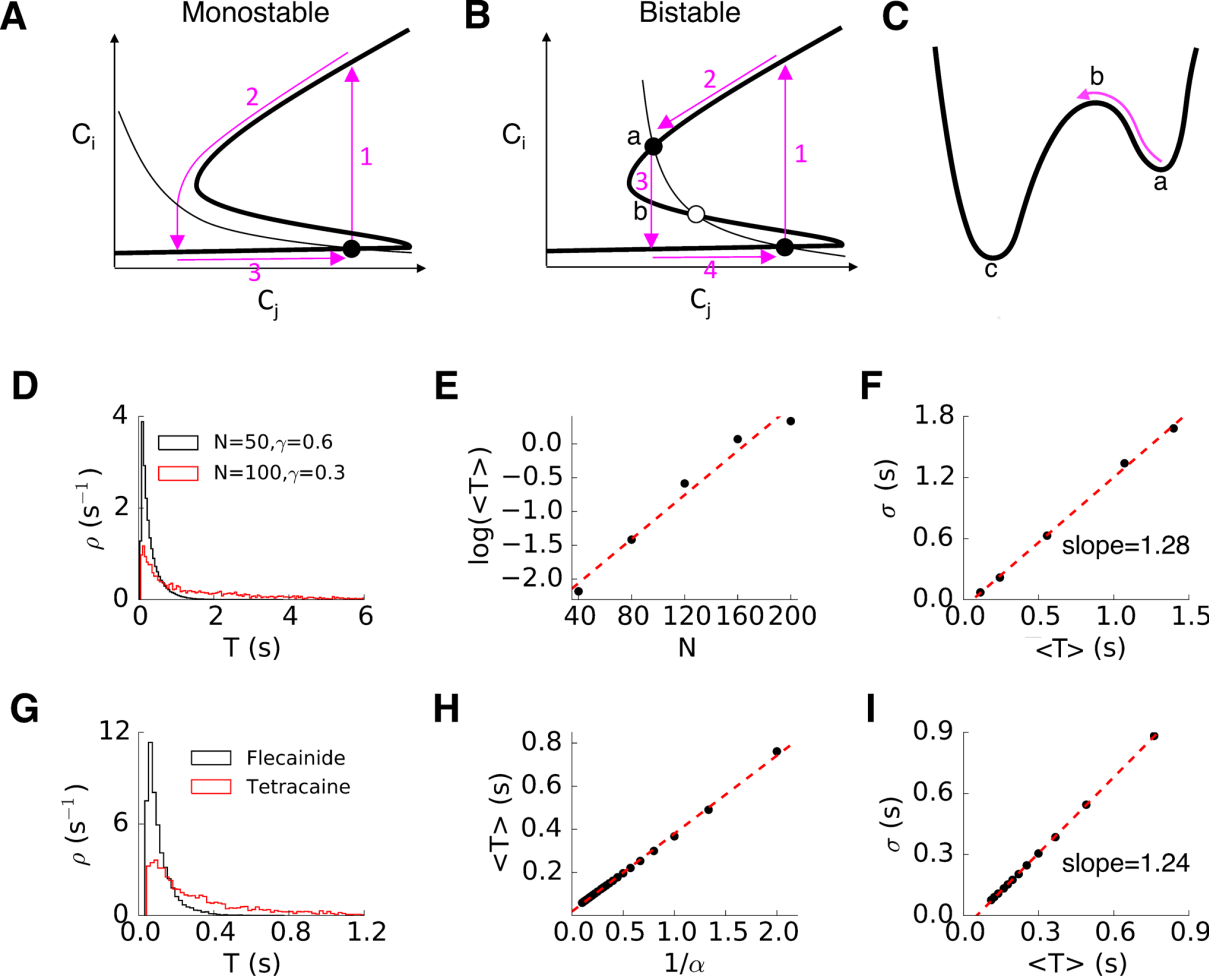
Determinants of spark duration. **A**. Schematic plot illustrating the dynamical process of a normal spark. The black lines are the nullclines as in Fig 5. Arrow 1: Collective opening of RyRs via CICR, Arrow 2: SR depletion and/or luminal Ca regulation of RyRs shutting off CICR, terminating the spark. Arrow 3. SR Ca reuptake. **B**. Schematic plot illustrating the dynamical process of a long-lasting spark. Same as A but SR depletion does not cause SR Ca to be low enough to shut off CICR, and the termination of the spark stochastic transition across the potential barrier (arrow 3). **C**. Schematic plot of potential wells illustrating Kramers scape process. **D**. Spark duration distributions for a 50% reduction of the Ca release flux strength by either reducing γ from 0.6 to 0.3 or reducing N from 100 to 50. **E**. <T> versus N for changing γ and N while keeping γN = 60. α = 1 and β = 1. **F**. σ versus <T> for the same simulations in E. **G**. Spark duration distributions under tetracaine (α = 1 and β = 1) and flecainide (α = 4 and β = 4). **H**. <T> versus 1/α for changing α and β while keeping α/β = 1. **I**. σ versus <T> for the same simulations in H.

Based on the dynamical analysis, when the deterministic limit has one stable solution (Fig 5A), normal sparks occur. Random opening of RyRs can trigger CICR, causing most of the RyRs to open (arrow 1 in Fig 8A), which then depletes the SR (arrow 2 in Fig 8A). When the SR Ca is too low, CICR cannot be maintained either due to the release flux becoming too small or because of luminal Ca-dependent regulation of RyRs. The release stops and SR is refilled by the SERCA pump to the steady state (arrow 3 in Fig 8A). This is a typical excitable transient process in which the duration of the spark is determined by the duration of the excitable transient corresponding to the time it takes to deplete the JSR Ca below the critical level to sustain CICR. This explains why the duration of normal sparks exhibits a bell-shaped distribution. For long-lasting sparks, the deterministic limit has two stable states (Fig 5B). CICR cannot automatically shut off but rather is maintained at the high stable state. Termination of the release is a stochastic transition across a potential barrier (arrow 3 in Fig 8B), i.e., the classical Kramers’ escape process [35] and the spark duration is then the first-passage time across the barrier. To understand what determines the duration of the long-lasting sparks, we use the Langevin equation to describe the stochastic opening of the RyRs in a CRU as [36]:

$$\frac{dp}{dt}=k_{o}(1-p)-k_{c}p+\sqrt{\frac{k_{o}(1-p)+k_{c}p}{N}}\xi (t)$$ (10)

where p is the open probability of RyR and N is the number of RyRs in a CRU. *ξ*(*t*) is a Gaussian white noise with < *ξ*(*t*) > = 0 and < *ξ*(*t*)*ξ*(*t*') > = *δ*(*t*−*t*'). *k*_o_ and *k*_c_ are the transition rate constants with *k*_o_ being a function of Ca concentrations in the dyadic space (c_p_) and JSR (c_j_) and *k*_c_ = β (see Eqs 26 and 27 in Methods). Since c_p_ changes quickly due to the small volume of the dyadic space, one can represent it by a function of p using a quasi-steady state approximation [14,37,38]. Eq 10 can then be rewritten as

$$\frac{dp}{dt}=f(p,c_{j})+\sqrt{\varepsilon (p,c_{j})}\xi (t)$$ (11)

where

$$f(p,c_{j})=k_{o}(1-p)-k_{c}p=\alpha h(c_{j}){\left(\frac{v_{p}c_{s}\;/\;\tau_{p}+\gamma Npc_{j}}{v_{p}\;/\;\tau_{p}+\gamma Np}\right)}^{2}(1-p)-\beta p$$ (12)

and *h*(*c*_j_) is a Hill function describing luminal Ca-dependent regulation (see Methods), *v*_p_ is the dyadic space volume, *τ*_p_ is the diffusion time constant from the dyadic space to sub-membrane space, and *c*_s_ is the Ca concentration in the sub-membrane space (see Restrepo et al [14]). We further assume that the noise strength can be approximated using the steady-state values, i.e.,

$$\varepsilon =\frac{2p_{s}(1-p_{s})}{N\tau }$$ (13)

where *p*_s_ is the steady-state open probability:

$$p_{s}=\frac{k_{o}}{k_{o}+k_{c}}=\frac{1}{1+k_{c}/k_{o}}$$ (14)

and *τ* is the relaxation time of the RyRs:

$$\tau =\frac{1}{k_{o}+k_{c}}$$ (15)

With a constant noise strength approximation, the transition rate across the potential barrier is [39]:

$$r_{k}={\left(2\pi \right)}^{-1}\sqrt{U"(p_{a},c_{j})\left|U"(p_{b},c_{j})\right|}\;\text{exp}[-\frac{U(p_{b},c_{j})-U(p_{a},c_{j})}{\varepsilon }]$$ (16)

where *U*(*p*, *c*_*j*_) = −∫*f*(*p*, *c*_*j*_)*dp* is the potential function, U(p_a_,c_j_) the potential valley, U(p_b_,c_j_) the potential barrier (see schematic plot in Fig 8C), and $U"(p,c_{j})=\frac{\partial^{2}U(p,c_{j})}{\partial p^{2}}$.

The spark duration, T, is approximately the first-passage time of the system across the barrier, whose distribution can be obtained as follows. At time T, the total probability, q(T), that the system still stays in the potential valley obeys *dq*(*T*) / *dT* = −*r*_*k*_ *q*(*T*), and the total probability that the system has crossed the barrier is then *P*(*T*) = 1 − *q*(*T*). The escape rate at time T is *ρ*(*T*) = *dP*(*T*) / *dT*, which gives rise to:

$$\rho (T)=r_{k}\;\text{exp}(-r_{k}T)$$ (17)

and the average is

$$<T>=\int_{0}^{\infty }T\rho (T)dT=1/r_{k}$$ (18)

The standard deviation of exponential distribution is: *σ* = < *T* >. Therefore, the duration of long-lasting sparks exhibits an exponential distribution. Our simulation results and the experimental observations agree with this theoretical prediction.

Based on the Kramers’ theory (Eqs 16–18), the spark duration and its variance are determined by the noise strength and the height of the potential barrier, which depend on the SR Ca content, the RyR open probability, the RyR cluster size, as well as the relaxation time of the RyRs. The theory can explain qualitatively the results shown in Fig 4 that reducing the Ca release flux by different ways can promote long-lasting sparks but results in different average spark durations for the same change of release flux strength. For example, simulations showed that reducing the single channel conductance of RyR (γ) yields longer averaged spark duration than reducing the number (N) of RyRs in a CRU (Fig 8D, and compare the results in Fig 4C and4D), even though those two interventions reduce the Ca release flux by the same amount. This is because reducing N also increased the noise strength, which resulted in shorter spark durations for the same reduction in Ca release flux. Based on the theory above, if one changes N and γ while keeping γN = *constant*, there will be no change in the driving force according to Eq 12 and thus no change in the potential barrier. According to Eq 13, the noise strength will be changed, inversely proportional to N. Therefore, using Eqs 16 and 18, one obtains that the average spark duration increases exponentially with N, i.e.,

<T>∝exp(μN) (19)

Therefore, increasing the number of RyRs in a CRU while keeping the release flux unchanged, although reducing the noise strength, increases the spark duration and its variance. To confirm this theoretical prediction (Eq 19), we carried out simulations by changing N and γ while keeping γN = *constant* in the ventricular myocyte model, and the average spark durations from the simulations indeed increased with N over a 5-fold change in N (Fig 8E). The standard deviation is slightly greater than <T> (Fig 8F), which may be due to the fact that the spark distributions are not precisely exponential in our simulations.

In Fig 4A and 4B, RyR open probability was reduced by either reducing α (tetracaine) or increasing β (flecainide) to cause long-lasting sparks. For the same α/β, the RyR open probability was the same, but the spark duration was shorter in the case of increasing β than the case of decreasing α (one example shown in Fig 8G). If one varies α and β while maintaining the same α/β ratio, then both the driving force in Eq 12 (and thus the potential U) and the noise strength in Eq 13 scale with α. Using Eqs 16 and 18, one has

<T>∝1/α (20)

Using the ventricular myocyte model, Fig 8H shows that changing α and β while keeping α/β = 1 produces a linear relation between <T> and 1/α, which agrees with the theoretical prediction. In these simulation results, the standard deviation is slightly greater than <T> (Fig 8I).

## Discussion

In this study, we combined mathematical analysis and computer simulation to investigate the mechanism underlying the transition from normal brief sparks to long-lasting sparks. We showed that in addition to CICR, CRU coupling and release competition in a CRU network play important roles in spark dynamics. Specifically, normal brief sparks occur when the CRU network is monostable and excitable, while long-lasting sparks are associated with multiple metastable attractors of the CRU network, with each attractor corresponding to a different spatial firing pattern of sparks. When the CRU firings are frequent, decreasing RyR open probability reduces the spark frequency, and thus there are more unfired CRUs to supply Ca to the fired CRUs to avoid Ca depletion in the JSR below the critical level which terminates the spark, thereby promoting long-lasting sparks. Decreasing RyR open probability further tends to suppress long-lasting sparks by weakening CICR. Due to the competition of CICR and SR Ca depletion, an optimal range of RyR open probability exists for long-lasting sparks to occur, such that depending on the starting conditions, long-lasting sparks can be induced by either increasing or reducing RyR open probability. This explains the experimental observations that long-lasting sparks can be promoted by increasing RyR open probability or decreasing the RyR open probability in different experiments. Based on previous modeling studies [31,32] and the present study, normal sparks are excitable transients, while long-lasting sparks are persistent firings which terminate via stochastic transitions across a potential barrier, a typical Kramers’ escape process. The duration of a long-lasting spark is the first passage time of the Kramers’ escape process, exhibiting an exponential distribution determined by the barrier height and noise strength. Our mathematical analysis using the Langevin equation reveals how the duration of the long-lasting sparks is modulated differently by different ways of altering the Ca release flux strength.

### Mechanisms for the transition from brief sparks to long-lasting sparks

It has been hypothesized that agents (e.g., FK506, rapamycin, and ryanodine) that increase RyR open probability promote long-lasting sparks by inducing long subconductance open states of RyRs [20,21]. However, long-lasing sparks have also been observed after tetracaine, Mg^2+^, or ruthenium red which reduce RyR open probability but do not increase RyR open time [25–27]. In our simulation, the average RyR open time (= 1/β) is ~ 1 ms, while the average spark duration can be several hundred milliseconds or seconds. Therefore, long-lasing sparks cannot be explained solely by the single channel properties of individual CRUs.

The mechanisms of long-lasting sparks have been investigated both theoretically and computationally using single CRU models. Sobie et al [30] were the first to simulate long-lasting sparks using a single CRU model in which they showed that decreasing the coupled gating of RyRs (which equivalently increased the RyR open probability) promoted long-lasting sparks. Hinch [31] used theoretical analysis to show that normal brief sparks are stochastic events of a system whose deterministic limit is a monostable excitable system, while long-lasting sparks are stochastic events of a system whose deterministic limit is a bistable system. He also derived analytical formulism of spark duration distributions for both normal and long-lasting sparks. In a more recent study [32], Stern et al revisited the mechanisms of Ca spark termination, in particular the effects of Ca diffusion between NSR and JSR, and showed that long-lasting sparks are metastable solutions of a CRU which is potentiated by increased RyR Ca sensitivity, increased RyR open probability, or increased Ca diffusion from NSR to JSR. All these previous studies and the theoretical analysis in the present study show that, when using a single CRU model, the transition from normal brief sparks to long-lasting sparks is promoted by increasing RyR open probability, which cannot explain the experimental observation that reducing RyR open probability also promotes long-lasting sparks.

Although Ca sparks are firings of individual CRUs, they are observed experimentally not in isolated CRUs but in a coupled network of CRUs. In a CRU network, CRUs are coupled via Ca diffusion in the NSR and cytosol. Thus CRU firings are affected by and also affect the neighboring CRUs. In a simulation study using a one-dimensional chain of coupled CRUs by Gaur and Rudy [40], long-lasting openings was observed with reduced Ca flux due to impaired luminal Ca sensor and buffering, agreeing with the observation that reducing RyR open probability induces long-lasting sparks, but the underlying mechanism was not investigated. In the present study, by focusing on networks of diffusively coupled CRUs, our analysis sheds new light on the mechanisms of long-lasting sparks, thereby helping to unify seemingly contradictory experimental observations. Specifically, in the deterministic limit of vanishing channel stochasticity, multiple stable solutions can co-exist in a coupled CRU network, exhibiting periodic and random spatial patterns of continuously firing CRUs surrounded by quiescent non-firing ones. In the real or model cells, sparks fire randomly, and the occurrence of long-lasting sparks is a random pattern selection process among the multiple solutions of the CRU network that become metastable in the presence of noise. When the spark probability is high, the system selects the uniform solution, resulting in brief sparks. When the spark probability becomes lower, the system selects non-uniform solutions, resulting in long-lasting sparks maintained by intra-SR diffusion of Ca from unfired neighboring CRUs. When the spark probability becomes very low, no multi-metastability occurs in the CRU network, again resulting in brief sparks only. Therefore, long-lasting sparks can be generally understood to result from two competing processes: spark frequency controlled by RyR open probability and the formation of multi-metastable firing patterns of the CRU network promoted by increased diffusive coupling. Due to this competition, long-lasting sparks occur in the intermediate range of RyR open probability, explaining why reducing or increasing RyR open probability can induce long-lasting sparks in different experiments.

Since reducing RyR open probability or the Ca release flux may cause an increase in SR Ca load (Fig 4), the question arises as to whether the increase of SR Ca load is responsible for the transition from normal sparks to long-lasting sparks. Based on the theoretical analysis (e.g., the nullcline analysis in Fig 5 or Eq 7), increasing the SR Ca load (or increasing the total Ca) will potentiate bistability by moving the c_j_-nullcline upwards and thus promote long-lasting sparks. However, reducing the Ca release flux will also weaken CICR and move the c_i_-nullcline upwards, making it difficult to sort out whether increased SR Ca load or weaker CICR plays a dominant role in sustaining long-lasting sparks using the single CRU analysis. At the transition from short sparks to long-lasting sparks obtained by decreasing the Ca release flux in the simulations (cf. Fig 4), the SR Ca load only increased slightly (see, in particular, the cases shown in Fig 4C and 4D). However, in those simulations, the spark frequency decreased quickly, indicating that there were many unfired CRUs that could provide Ca to avoid the Ca in the JSR of the firing CRUs to be depleted below a critical value for persistent firings. In fact, Zima et al [27] showed that increasing SR load alone (by 36%) without blocking RyR open probability would not produce long-lasting sparks. If the increase in SR load alone were responsible for the induction of long-lasting sparks, all CRUs would fire long-lasting sparks. However, only a very few of the CRUs exhibited long-lasting sparks in our simulations as well as in experiments [27]. We conclude that in both our simulations and experiments, increase in SR load might contribute to the induction of long-lasting sparks, but that the reduction in spark frequency is likely to be the major contributor by allowing Ca to diffuse from unfired to fired CRUs so as to prevent Ca depletion below the critical level for termination of CICR.

In real ventricular myocytes, the RyR clusters are heterogeneous [41], which may also contribute to the induction of long-lasting sparks. Since the number of RyRs and their spatial distribution within a CRU may vary, different CRUs may exhibit different CICR properties. As a result, some CRUs may have a higher likelihood to fire and hence preferentially drain Ca from unfired CRUs, making those unfired CRUs even less likely to fire. The occurrence of long-lasting sparks may also be affected by the heterogeneity of the NSR network in which a given CRU may functionally link different numbers of CRUs in different regions. As shown by Zima et al [26], CRUs with fewer linked neighbors tended to fire short sparks while CRUs with more neighbors tended to fire long-lasting sparks, indicating that the supply of Ca from the neighboring unfired CRUs is important for maintaining the long firings. Due to the CRU heterogeneity in the real system, both brief and long-lasting sparks can co-exist in the same cell, which may account for the modularity of spark duration distributions seen in experiments [27]. However, while although heterogeneity alone can explain why some CRUs preferentially exhibit long-lasting sparks, it cannot explain why reducing RyR open probability promotes long-lasting sparks.

We need to point out that whether increasing RyR open probability or decreasing RyR open probability promotes long-lasting sparks depends on the conditions of the system, such as temperature, intracellular Na level, as well as the species. As shown in Figs 2 and 3, when the cell had a high SR load and low spark frequency, increasing RyR open probability promoted long-lasting sparks, but when the cell had a low SR load and high spark frequency, reducing RyR open probability promoted long-lasting sparks. Since the RyR properties and intracellular Na level that affect Ca load [42] may vary with species and diseases, such that in some cases, decreasing RyR open probability may promote long-lasting sparks, whereas in others, increasing RyR open probability may be required.

### Dynamical mechanisms of spark termination

The mechanism of Ca spark termination has been debated for decades and different biological causes and mechanisms have been proposed [43–47], including stochastic attrition [43], cytosolic Ca-dependent inactivation [43], allosteric coupling [30], luminal Ca-dependent inactivation [48,49], SR Ca depletion [30], and induction delay [50]. Theoretically, any of these mechanisms or a combination of several of them can terminate a Ca spark, but which of them exists in the real cell has been the subject of debate for decades. Although the exact biological causes remain unclear, two qualitatively distinct dynamical mechanisms can be distinguished from the basic nonlinear dynamics perspective of the present article. As illustrated in Fig 8A, a normal spark is an excitable transient of a stochastically excitable system. Randomly opening one or more RyRs or LCCs results in an increase of Ca in the dyadic space, which can elicit CICR to cause RyRs to open. The CICR keeps the RyRs open which depletes the JSR. When the JSR Ca is depleted below a critical level, the CICR stops because: 1) the Ca in the JSR is so low so that the Ca flux through the RyRs cannot maintain the Ca level in the dyadic space to sustain the CICR; and 2) Reduced SR Ca causes inactivation of the RyRs so that the number of open RyRs decreases, further reducing the Ca flux required to sustain CICR. As CICR declines, all RyRs close and the spark terminates. However, when the JSR Ca cannot be depleted to the critical level, CICR is sustained (Fig 8B). Termination of CICR is due to stochastic fluctuations of RyR openings that cause the system to cross the potential barrier (Fig 8C), i.e., due to stochastic fluctuations (or stochastic attrition [32]), the number of open RyRs at a certain time is below a critical number so that the Ca flux is not strong enough to sustain CICR. Since termination of a normal spark is via JSR Ca depletion, its duration is determined by the Ca release flux strength. The stochastic channel noise causes the spark duration to fluctuate around its mean, which is not highly variable. In contrast, a long-lasting spark (Fig 8B) is a stochastic bistable switch whose dynamics follow Kramers’ escape theory. The spark duration in this case exhibits an exponential distribution with a large variation. As shown in our theoretical analysis (Eqs 13–18), the average spark duration strongly depends on the relaxation time (τ), open probability of the RyRs, and the size of RyR clusters and thus on the specific ways of changing the Ca release flux strength. The theoretical predictions agree well with the behaviors observed in simulations of the detailed ventricular myocyte model. Therefore, the two types of sparks are caused by two distinct dynamical mechanisms, and the transition from brief to long-lasting sparks is a transition from monostability to bistability or multistability.

### Limitations

**Fig 9:**
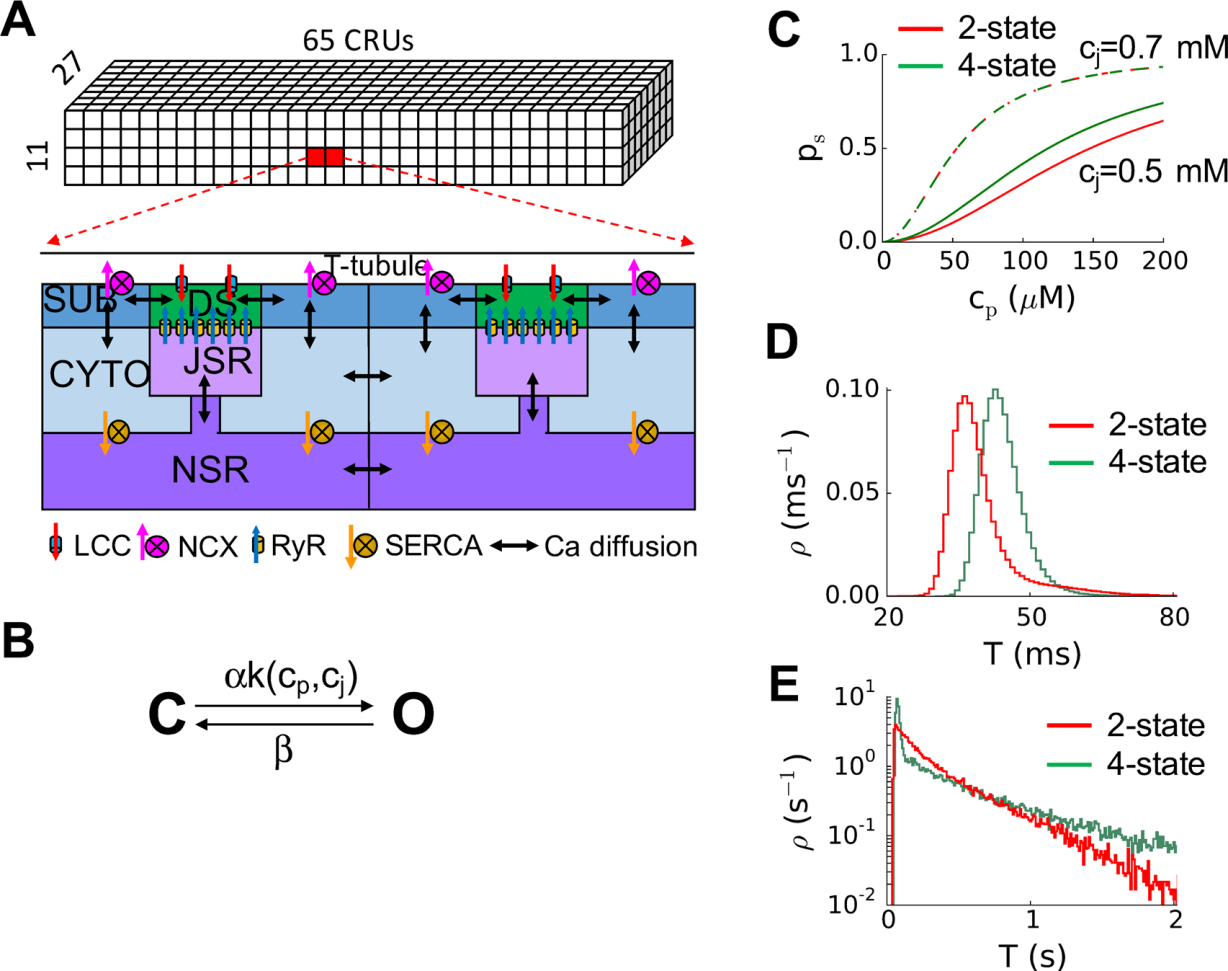
Computational models. **A**. Schematic plot of the ventricular myocyte model and the coupling between different compartments and CRUs. DS—dyadic space, SUB—sub-membrane space, CYTO-cytosolic space, NSR—network SR, and JSR—junctional SR. **B**. The 2-state RyR model. **C**. RyR open probability versus c_p_ for the 2-state and 4-state model for two different JSR Ca concentrations. The open probability is lower for the 2-state model when c_j_ = 0.5 mM but when c_j_ = 0.7 mM, the open probabilities of the two models are almost identical. **D**. Spark duration distributions for the 2-state and 4-state model for normal brief Ca sparks (α = 4 and β = 1). **E**. Spark duration distributions for the 2-state and 4-state model for long-lasting sparks (α = 1 and β = 1). The same α scaling factor was used for the 4-state model to change RyR open probability, i.e., we multiplied k_12_ and k_43_ by the factor α in the original 4-state model. The simulations for D and E were done using the whole-cell model.

Several limitations of the present study need to be pointed out. The CRU network is a homogenous model, while the real CRU network is highly heterogeneous [41]. We used a very simple model of the spatial structure of a CRU, while spatially detailed models of CRUs have been developed recently [50–53], and a new mechanism of spark termination was shown in such detailed models [45,50]. Adding the detailed structural CRU information may reveal additional mechanistic insights into long-lasting sparks. Finally, the detailed spark dynamics may also depend on the specific RyR model used. We used a 2-state RyR model that incorporated the luminal Ca regulation of RyR, simplified from the original 4-state model. As we showed in Fig 9, the two models exhibited similar spark dynamics despite small quantitative difference. Nevertheless, the dynamical mechanisms of the transition from brief to long-lasting sparks revealed in the present are likely generic ones that are applicable to Ca spark dynamics in the real systems.

## Methods

### Ventricular myocyte model

We used the ventricular myocyte model developed by Restrepo et al [14], which contains a three-dimensional network of 19,305 (65x27x11) CRUs (Fig 9A) with CRU spacing being 1.84 μm in the longitudinal direction and 0.9 μm in the transverse direction, corresponding to a cell of dimension ~120×25×10 *μ*m^3^. The CRUs are coupled via Ca diffusion in the cytosolic space and SR. Each CRU contains five sub-volumes (lower panel in Fig 9A): NSR, JSR, dyadic space or proximal space, sub-membrane space, and cytosolic space. Each CRU has a cluster of 100 RyR channels simulated using random Markov transitions. The Ca concentrations in the five compartments for an arbitrary CRU are described by the following differential equations:

$$\frac{{\text{dc}}_{\text{i}}}{\text{dt}}=\beta_{\text{i}}\left({\text{c}}_{\text{i}}\right)\;\left({\text{I}}_{\text{dsi}}\frac{{\text{v}}_{\text{s}}}{{\text{v}}_{\text{i}}}-{\text{I}}_{\text{up}}+{\text{I}}_{\text{leak}}-{\text{I}}_{\text{TCi}}+{\text{I}}_{\text{ci}}+{\text{I}}_{\text{b}}\right)$$ (21)

$$\frac{{\text{dc}}_{\text{s}}}{\text{dt}}=\beta_{\text{s}}\left({\text{c}}_{\text{s}}\right)\;\left({\text{I}}_{\text{dps}}\frac{{\text{v}}_{\text{p}}}{{\text{v}}_{\text{s}}}+{\text{I}}_{\text{NCX}}-{\text{I}}_{\text{dsi}}-{\text{I}}_{\text{TCs}}\right)$$ (22)

$$\frac{{\text{dc}}_{\text{p}}}{\text{dt}}=\beta_{\text{p}}\left({\text{c}}_{\text{p}}\right)\;\left({\text{I}}_{\text{rel}}+{\text{I}}_{\text{Ca},\text{L}}-{\text{I}}_{\text{dps}}\right)$$ (23)

$$\frac{{\text{dc}}_{\text{nsr}}}{\text{dt}}=\left({\text{I}}_{\text{up}}-\;{\text{I}}_{\text{leak}}\right)\frac{{\text{v}}_{\text{i}}}{{\text{v}}_{\text{nsr}}}-{\text{I}}_{\text{tr}}\frac{{\text{v}}_{\text{jsr}}}{{\text{v}}_{\text{nsr}}}+{\text{I}}_{\text{cnsr}}$$ (24)

$$\frac{{\text{dc}}_{\text{jsr}}}{\text{dt}}=\beta_{\text{jsr}}\left({\text{c}}_{\text{jsr}}\right)\;\left({\text{I}}_{\text{tr}}-\;{\text{I}}_{\text{r}}\frac{{\text{v}}_{\text{p}}}{{\text{v}}_{\text{jsr}}}\right)$$ (25)

where c_i_ is the free Ca concentration in the cytosolic space, c_s_ is the free Ca concentration in the sub-membrane space, c_p_ is the free Ca concentration in the proximal space (dyadic space), c_jsr_ is the free Ca concentration in the junctional SR, and c_nsr_ is the free Ca concentration in the network SR. The *β* terms account for instantaneous buffers in corresponding compartments using the rapid buffering approximation [54]. I_NCX_ is Na-Ca exchange flux and I_Ca,L_ is the L-type Ca flux (Note: the same symbols as the membrane ionic currents were used but they are ion fluxes). I_up_ is the SERCA uptake current representing total flux into the NSR, I_leak_ is the leak current from NSR to cytosol, and I_rel_ is the total Ca efflux from the JSR. I_dsi_, I_dps_ and I_tr_ are the diffusion currents from adjacent compartments, I_TCi_ and I_TCs_ are the troponin C dynamic buffering currents in cytosol and sub-membrane spaces, and I_ci_ and I_cnsr_ are the diffusive currents between neighboring CRUs in the corresponding compartments. v_s_, v_i_, v_p_, v_nsr_, and v_jsr_ are the volumes of the sub-membrane space, cytosolic space, dyadic space, NSR, and JSR, respectively.

We made the following changes (and the small corrections stated in Restrepo and Karma [55]) from the original model [14]:

1. Since our aim is to investigate the behaviors of Ca sparks, not the whole-cell Ca and action potential dynamics, we clamped the voltage at V = -80 mV. Because the voltage is clamped -80 mV, no L-type Ca channel opens and thus no Ca entry, intracellular Ca level decreases with time due to NCX. To maintain intracellular Ca homeostasis, we added a background Ca current: I_b_ = g_b_(V-E_Ca_) with g_b_ = 0.001149 mS/cm^2^. $E_{Ca}\;=\frac{RT}{2F}ln\frac{{\left[Ca\right]}_{o}}{c_{i}}$ is the reversal potential, in which R is the gas constant, T is temperature, F is the Faraday constant, and [Ca]_o_ is the extracellular Ca concentration.
2. To facilitate analytical treatment, we simplified the original 4-state RyR model into a 2-state model (Fig 9B). We incorporated the luminal Ca regulation of RyRs by changing the closed-to-open rate constant to
  $$k_{o}=\alpha k(c_{p},c_{j})=\alpha K_{u}c_{p}^{2}\frac{c_{j}^{h}}{c_{j}^{h}+c_{jth}^{h}}$$ (26)
  and kept the open-to-closed rate unchanged, i.e.,
  $$k_{c}=\beta$$ (27)
  In Eq 26, *c*_p_ is the Ca concentration in the dyadic space, *c*_j_ is the Ca concentration in junctional SR, and c_jth_ is the dissociation constant for luminal Ca regulation of RyRs, which was set as c_jth_ = 610 μM. *h* = 10 is the Hill coefficient. *K*_*u*_ = 0.00038 μM^-2^ ms^-1^ is a rate constant that was unchanged from the original 4-state model ($k_{o}=k_{12}=K_{u}c_{p}^{2}$ in the original model). The 2-state model has the same open probability as the 4-state model when c_j_>c_jth_ but has a smaller open probability when c_j_<c_jth_ (Fig 9C). This is because the original model has a more complex luminal Ca regulation of RyRs than the simple Hill function as in Eq 26. In Eq 26, we added a scaling factor α to change the RyR open probability. α = 4 and β = 1 ms^-1^ were used for control. Based on experimental observations [33], tetracaine reduces RyR open probability by increasing the closed time while flecainide reduces RyR open probability by decreasing the open time, we reduced α to model the effects of tetracaine and increase β to model the effects of flecainide. Although the 2-state model is not the same as the 4-state model, the resulting spark dynamics are qualitatively the same. Fig 9D plots a histogram of spark duration for normal brief sparks, showing that the distributions are similar except that the spark duration is about 10 ms shorter for the 2-state model. Reducing α from 4 to 1 resulted in long-lasting sparks using both models (Fig 9E) with a quantitative difference in spark duration distribution.
3. Since the goal of this study is to investigate the dynamics of individual sparks, we needed to avoid Ca waves in the model. In the original model by Restrepo et al [14], the sub-membrane spaces of adjacent CRUs are coupled via Ca diffusion. Since the Ca concentration in the sub-membrane space is relatively high and it can easily cause waves as Ca load increases due to this coupling (Note: the sub-membrane space coupling may “shortcut” the adjacent CRUs, in particular for the ones in the longitudinal direction, to promote Ca waves, which is a limitation of the original model). Therefore, we removed the Ca diffusion between sub-membrane spaces of adjacent CRUs while keeping the other intra- and inter-CRU Ca diffusion couplings unchanged. In many experimental studies of Ca sparks, addition of Ca buffers, such as EGTA, was used to weaken the coupling between CRUs to prevent Ca waves [11,56].
4. We changed the volume of the junctional SR and the volume of the proximal space to be 150% and 70% of the original values, respectively. We also used a scaling factor γ to alter the single channel conductance of RyRs. γ = 0.6 was set as the control value.

The changed parameters from the original model [14] are listed in Table 1.

**Table 1:** Changed parameters from the original model by Restrepo et al [14].

Parameter	Value	Unit
ν_NaCa_	7	μM ms^−1^
v_p_	0.000882	μm^3^
v_jsr_	0.03	μm^3^
ν_up_	0.36	μM ms^−1^
J_max_	0.000882	μm^3^ ms^−1^
B_CSQN_	460	μM
[Na]_i_	12	mM

### Computer simulation methods

The RyRs were simulated using a stochastic simulation method described previously [38]. The time step for integration of the differential equation is 0.01 ms. The total simulation time is at least 100 s and data were collected after 10 s to ensure that the system reached its steady-state condition. Simulations were done using Graphic Processor Units (Nvidia Tesla K20) with CUDA and C language. The source code of the ventricular myocyte model is available at the following link, https://bitbucket.org/quslab/long-lasting-spark-model/src.
